# Validity of the Turkish version of the Oral Frailty Index-8 and its relationship with frailty, malnutrition, and sarcopenia in older adults

**DOI:** 10.55730/1300-0144.6137

**Published:** 2025-11-14

**Authors:** Süleyman Emre KOÇYİĞİT, Bilal KATİPOĞLU, Yılmaz ÖNAL, Kübra BAHAR BAYKAN, İlknur ŞEKER KAYA, Fatma Sena DOST, Özge DOKUZLAR, Esra ATEŞ BULUT, Ali Ekrem AYDIN, Derya KAYA, Ahmet Turan IŞIK

**Affiliations:** 1 Division of Geriatric Medicine, Department of Internal Medicine, Faculty of Medicine, Balıkesir University, Balıkesir, Turkiye; 2 Division of Geriatric Medicine, Balıkesir Atatürk City Hospital, Balıkesir, Turkiye; 3 Unit for Aging Brain and Dementia, Division of Geriatric Medicine, Department of Internal Medicine, Faculty of Medicine, Dokuz Eylül University, İzmir, Turkiye; 4 Division of Geriatric Medicine, Bursa Yüksek İhtisas Training and Research Hospital, Bursa, Turkiye; 5 Division of Geriatric Medicine, Adana City Hospital, University of Health Sciences, Adana, Turkiye; 6 Department of Geriatric Medicine, Department of Internal Medicine, Faculty of Medicine, On Dokuz Mayıs University, Samsun, Turkiye

**Keywords:** Oral frailty, frailty, validation study, malnutrition, sarcopenia

## Abstract

**Background/aim:**

The Oral Frailty Index-8 (OFI-8) is a reliable screening for oral frailty. In our study, while presenting the Turkish reliability and validity study of the OFI-8, we also aimed to establish a cut-off value for oral frailty in Turkish population.

**Materials and methods:**

Patients who presented to the outpatient clinic between January 2024 and January 2025 were evaluated. The test’s internal consistency and test–retest reliability were assessed. Divergent validity was evaluated using frailty scores, muscle strength, and nutritional status. A receiver operating characteristic (ROC) curve was generated to determine the optimal cut-off values for identifying frailty, malnutrition, and probable sarcopenia. Oral frailty status was then analyzed in relation to demographic characteristics, comorbidities, and geriatric syndromes. Regression analyses were performed to adjust for confounding factors.

**Results:**

In 162 patients, internal consistency was quantified with a Cronbach’s α of 0.728, and test–retest reliability was quantified with an intraclass correlation coefficient (ICC) of 0.961. Construct validity was supported by exploratory factor analysis, and divergent validity was confirmed through significant correlations with frailty, muscle strength, and nutritional status. A cut-off score of ≥ 5 on the OFI-8 was identified for predicting physical frailty, malnutrition, and probable sarcopenia: for frailty status, the area under the curve (AUC) was 0.75 [95% confidence interval (CI) 0.67–0.82; p < 0.001], sensitivity 0.85 and specificity 0.56; for probable sarcopenia, AUC was 0.68 (95% CI 0.60–0.77; p < 0.001), sensitivity 0.71 and specificity 0.52; and for nutritional status, AUC was 0.76 (95% CI 0.68–0.84; p < 0.001), sensitivity 0.86 and specificity 0.55. Independent of confounding factors, probable sarcopenia, physical frailty, polypharmacy, malnutrition, and falls remained significantly associated with oral frailty (OFI-8 score ≥5).

**Conclusion:**

OFI-8 demonstrates good validity and reliability for detecting oral frailty in Turkish older adults, supporting early diagnosis and intervention to address better related conditions such as frailty, probable sarcopenia, and malnutrition.

## 1. Introduction

Countries worldwide are experiencing a rapid demographic transition, and the World Health Organization (WHO) projects that the proportion of adults aged ≥60 years will reach approximately 20% by 2050. As populations age, the burden of age-related conditions, including frailty, is expected to rise [1]. Therefore, it is not surprising that the proportion of age-related problems will increase in an aging society. Since the risk of chronic and multiple diseases increases in older adults, the frequency of geriatric syndromes will also increase. One of these is the risk of frailty associated with age [2]. Frailty is defined as increased sensitivity to stressors resulting from decreased physiological reserve [2]. It is essential to screen for frailty, a dynamic, biopsychosocial process, in geriatric practice [3]. In addition, frailty is associated with adverse health outcomes such as falls, cognitive impairment, long-term care needs, disability, and even death [4,5]. It is widely documented that malnutrition and sarcopenia, other geriatric syndromes, are closely related to frailty and share common pathophysiological mechanisms [6]. Malnutrition and sarcopenia are associated with adverse outcomes, just as in frailty, and have a risk of morbidity and mortality in older adults [6,7].

Oral health refers to the comprehensive health of the oral cavity, where chewing, swallowing, tasting, and salivating functions occur [8]. Oral health is crucial for maintaining physical and mental well-being in older adults and serves as a primary indicator of overall health during the aging process [9]; however, oral health problems become more prevalent with age [10]. Remarkably, impaired oral health increases the risk of frailty, or oral health status can be negatively affected in frail older adults, suggesting a bidirectional relationship between oral health and frailty. Furthermore, poor oral health is associated with frailty as well as malnutrition, sarcopenia, and mortality [11].

In fact, frailty, due to its multifaceted and multisystemic nature, is an umbrella term that encompasses distinct concepts such as physical frailty, cognitive frailty, and social frailty [12]. Moreover, oral frailty, as defined by Tanaka, is a different type of frailty characterized by a progressive decline in oral functions associated with age, and it is a significant predictor of physical frailty, malnutrition, disability, sarcopenia, and mortality [13]. Given the reversibility of oral frailty, early recognition is crucial to perform the necessary interventions. For this purpose, many screening methods, such as the Oral Frailty Index-6 and the Oral Frailty Checklist (5-item), have been developed for clinical practice [13,14]. Oral Frailty Index-8 (OFI-8) items, developed by Tanaka in 2021 [15], roughly assess tooth loss, poor oral function, oral health-related behaviors, and decreased social participation [15], being useful for clinical practice. Furthermore, while oral health-related behaviors may vary significantly across different societies, no oral frailty screening tool has been developed specifically for Turkish older adults to date. Additionally, as far as we are concerned, the OFI-8 items have not yet been validated in Turkish. Moreover, existing oral health instruments, such as the Oral Health Impact Profile, focus on oral health-related quality-of-life domains and do not assess age-related declines in oral function or the frailty-related multidimensional mechanisms that the OFI-8 captures; therefore, they are insufficient for oral frailty screening [16]. Consequently, this study aimed to assess the validity and reliability of the Turkish version of the OFI-8 and to investigate its relationship with physical frailty, malnutrition, and probable sarcopenia in Turkish older adults. We hypothesized that the Turkish version of the OFI-8 would demonstrate good psychometric properties and correlate significantly with established markers of frailty, malnutrition, and sarcopenia.

## 2. Methods

### 2.1. Study population

All patients who applied to our geriatrics clinic as outpatients between January 2024 and January 2025 were evaluated. The participants were consecutive outpatients who underwent a comprehensive geriatric assessment by an experienced geriatrician at a tertiary hospital.

**Figure 1:**
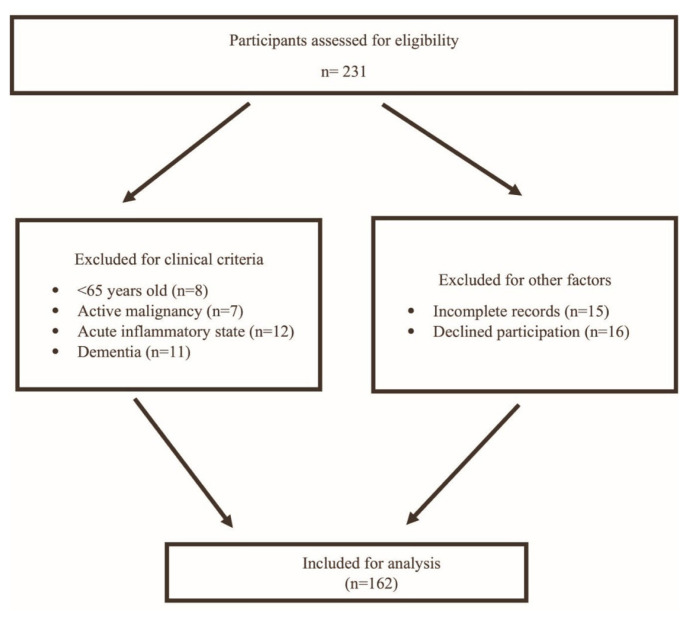
Flow diagram of participant selection and inclusion process.

Patients who had a history of severe illness that might negatively affect general and oral health status (such as an acute cerebrovascular event, gastrointestinal bleeding, severe sepsis, acute renal injury, acute coronary syndrome, acute liver failure, active malignancy (taking chemotherapy or radiotherapy), or acute respiratory failure) and those aged <65 years were excluded from the study. In addition, patients who had a diagnosis of Clinical Dementia Rating scale (CDR) ≥ 2 dementia and had Mini-Mental State Examination (MMSE) score <18 were excluded from the study, because patients with moderate to advanced dementia cannot express their own thoughts about oral health issues. In addition, the original OFI-8 questionnaire developed by Tanaka et al. included MMSE <18 as an exclusion criterion. The participant selection process is summarized in Figure 1.

An experienced geriatrician assessed and recorded the participants’ demographic characteristics including age, sex, marital status, living environment, comorbidities (hypertension, diabetes mellitus, peripheral arterial disease, chronic cardiac disease including congestive heart disease, atrial fibrillation and ischemic heart disease, osteoporosis and chronic lung disease status) and Charlson Comorbidity Index (CCI), geriatric syndromes including recurrent falls in a year (≥ 2), polypharmacy (≥5 medication use), urinary incontinence, sleep disorder and geriatric depression.

### 2.2. Comprehensive geriatric assessment

In the comprehensive geriatric assessment performed by geriatricians, MMSE was used for the cognitive evaluation, and the Yesevage 15-item Geriatric Depression Scale; Barthel Index for basic activities of daily living (ADLs); Lawton–Brody Index for instrumental ADLs; and Tinetti Performance-Oriented Assessment of Mobility and Timed Up and Go (TUG) for mobility evaluation [17].

### 2.3. Sample size calculation

The sample size power calculation was performed based on the expected Cronbach’s alpha. According to the rules-of-thumb for the minimum sample size to be included in the study and the rule of recruiting four to 10 participants for each variable, when a minimum of 10 participants were recruited for eight items with a dichotomous nature, it was estimated that a minimum of 80 patients would be recruited to ensure variance–covariance matrix stability [18]. In our study, 162 participants who met the inclusion criteria were recruited.

### 2.4. Translation procedure

Translating the OFI-8 into Turkish involved a five-step process: (1) the first step was to perform translation permission from the authors of the original questionnaire; (2) three native language experts made three independent translations who have clinical background into Turkish, and all translators were unaware of each other’s translations; (3) the translations were then analyzed by another researcher whose native language was Turkish, and a single text was created; (4) this consensus forward version was back-translated into English by two native language experts, and the back version and the original text were compared with the English translation; no items in the Turkish text required any changes after this step; and (5) the final text was administered to 10 patients to test whether there were any problems with administration; in the pilot test, none of the items were reported as confusing and no changes were needed. Although formal completion time was not recorded, all participants completed the questionnaire within a few minutes without needing assistance. An expert panel or Delphi consensus approach was not used. Instead, a forward–backward translation process with independent bilingual translators was applied (Table 1).

### 2.5. Diagnosis of physical frailty

Frailty status was assessed using the Fried Frailty Phenotype criteria, a widely accepted and validated method for identifying physical frailty in older adults. According to this model, five components were evaluated: unintentional weight loss (self-reported unintentional loss (reported by the patient or patients’ caregivers) of ≥4.5 kg within the past 12 months), exhaustion, weakness (measured by grip strength using a hand-held dynamometer cut-off values were adjusted for sex and body mass index (BMI), in accordance with the original criteria), slowness (assessed by measuring gait speed over a standardized distance (4 m), low physical activity. Participants meeting three or more of these criteria were classified as frail. Those with one or two criteria were considered nonfrail [19].

### 2.6. Assessment of malnutrition

Nutritional status was evaluated using the Short-Form Mini-Nutritional Assessment (MNA-SF), a validated screening tool widely used in geriatric populations to identify individuals at risk of malnutrition or who are already malnourished. The MNA-SF comprises six items addressing key domains: (1) food intake decline over the past 3 months due to loss of appetite, digestive problems, chewing or swallowing difficulties; (2) recent weight loss; (3) mobility; (4) psychological stress or acute disease in the past three months; (5) neuropsychological problems (e.g., depression or dementia); and (6) BMI or calf circumference if BMI is unavailable [20].

Each item is scored individually, with the total score ranging from 0 to 14. Based on the total score, nutritional status is categorized as follows:

- Normal nutritional status: 12–14 points
- At risk of malnutrition and malnourished: 0–11 points

### 2.7. Diagnosis of probable sarcopenia

Probable sarcopenia was diagnosed according to the European Working Group on Sarcopenia in Older People-2 diagnostic criteria. Accordingly, a diagnosis was made based on bilateral muscle strength measurements using a Jamar brand hand dynamometer [21]. Muscle strength was measured in three consecutive trials, and the highest value was recorded. Cut-off values for muscle strength were determined based on a validation study conducted in Türkiye, which defined probable sarcopenia as <14 kg in women and <28 kg in men [22].

### 2.8. The OFI-8

Oral frailty was evaluated using the OFI-8, a structured self-reported screening tool developed by Tanaka et al. The OFI-8 consists of eight items that address various aspects of oral health, function, and behavior [15]:

1. Have you found it more difficult to eat tough foods compared to 6 months ago?
2. Have you ever choked on your tea or soup?
3. Do you use dentures?
4. Do you often experience dry mouth?
5. Do you brush your teeth at least twice a day?
6. Do you visit the dentist at least once a year?
7. Can you chew hard foods such as pickled radish or dried squid?
8. Do you go out less frequently than you did last year?

Each item is scored dichotomously (Yes/No), with different weights assigned based on the predictive strength of each item:

- Items 1–3 (chewing difficulty, choking, denture use):
  Yes = 2 points, No = 0 points
- Items 4–8 (dry mouth, oral hygiene, dental visits, chewing hard foods, go out less frequently):
  Yes = 1 point, No = 0 points

### 2.9. Statistical analysis

In this study, nominal variables were shown with percentage (%), while continuous ones were expressed as mean + standard deviation. Internal consistency was assessed from Cronbach’s α value. Test–retest reliability was evaluated by administering the instrument twice at a 2-week interval to the same participants. The intraclass correlation coefficient (ICC) was computed to determine the temporal stability of the instrument. Construct validity was assessed via an exploratory factor analysis (EFA) using principal axis factoring with Promax rotation to examine the scale’s underlying factor structure. The Kaiser evaluated the adequacy of the data for factor analysis–Meyer–Olkin (KMO) measure and Bartlett’s test of sphericity. Divergent validity was evaluated by calculating Spearman correlation coefficients between the OFI-8 and the Fried Frailty Index MNA-SF, muscle strength, number of drugs, Yesevage 15-item Geriatric Depression Scale (GDS), MMSE, TUG test duration, and basic and instrumental ADLs. Receiver operating characteristic (ROC) curves are used to evaluate the validity of the OFI-8 for estimating physical frailty, probable sarcopenia, and nutritional status. Sensitivity, specificity, negative predictive value, and positive predictive value were calculated for each cut-off score. According to the total OFI-8 score cut-off value, those below the value were defined as the nonoral frailty group, and those above the value were defined as the oral frailty group. These two groups were then compared with respect to demographic characteristics, comorbidities, geriatric syndromes, and laboratory parameters. Nominal variables were compared using the chi-square test; if continuous variables were normally distributed, the Kolmogorov–Smirnov test was performed; otherwise, the Student’s t-test was performed. If continuous variables were not normally distributed, the Mann–Whitney U test was used. Then, binomial regression analysis was applied within oral and nonoral frailty groups, controlling for age, sex, and demographic characteristics related to geriatric syndromes. Multicollinearity among the variables was assessed using the Variance Inflation Factor (VIF) and tolerance values. No multicollinearity was detected. In all analyses, p < 0.05 was considered statistically significant. All statistical analyses were conducted using IBM SPSS Statistics version 22.0 (IBM Corp, Armonk, NY).

### 2.10. Ethics approval

This study received approval from the Ethics Committee under decision number 2024/11/73.

## 3. Results

### 3.1. Patient characteristics

Of the 162 patients, 63.4% were female, and the mean age was 77.4 ± 7.49 years. A total of 58.4% of the participants were primary school graduates, 62.1% were married, and 19.9% were living alone. The frequency of hypertension was 70.8%, type 2 diabetes mellitus was 38.5%, atherosclerotic heart disease was 26.7%, and chronic lung disease was 9.4%. The mean CCI value was 1.23+1.11. The rate of malnutrition risk was 26.7%, physically frail older individuals were 30.4%, probable sarcopenia was 37.3% and recurrent falls in the last year were 34.8%.

### 3.2. Test–retest reliability

The Cronbach’s alpha value for the OFI-8 was 0.728 [95% confidence interval (CI): 0.706–0.748], suggesting acceptable internal reliability. Item–total correlations ranged from 0.31 to 0.56, and Cronbach’s α if item deleted values remained close to the total α, indicating that no single item disproportionately affected overall internal consistency (Table 2a). High test–retest reliability was observed for the OFI-8, as reflected by a strong Spearman correlation (r = 0.922, p < 0.001) and an ICC of 0.961 (p < 0.001), demonstrating that the scale is stable over 14 days (Table 2b).

### 3.3. Construct validity

EFA was performed to assess the construct validity of the OFI-8 scale. The KMO measure was 0.75, and Bartlett’s Test of Sphericity was statistically significant (p < 0.001), indicating the data were suitable for factor analysis. Two factors had eigenvalues greater than 1, and the scree plot supported a two-factor solution. All items had factor loadings of ≥0.40 on at least one factor (Table 3).

### 3.4. Divergent validation

There was a negative moderate linear correlation between the OFI-8 score and MNA-SF, Barthel Index, Lawton–Brody Index, and muscle strength (r = −0.435, p < 0.001; r = −0.551, p < 0.001; r = −0.326, p < 0.001; r = −0.450, p < 0.001, respectively). In contrast, there was a statistically significant, moderate, positive correlation between the OFI-8 and the Fried Frailty Index score, TUG duration, and number of medications (r = 0.534, p < 0.001; r = 0.523, p < 0.001; r = 0.335, p < 0.001, respectively). There was a weak correlation between the OFI-8 and the CCI, and between the OFI-8 and the Yesevage 15-Item GDS. There was no significant correlation between MMSE score and the OFI-8 (Table 4).

### 3.5. ROC curve

**Figure 2:**
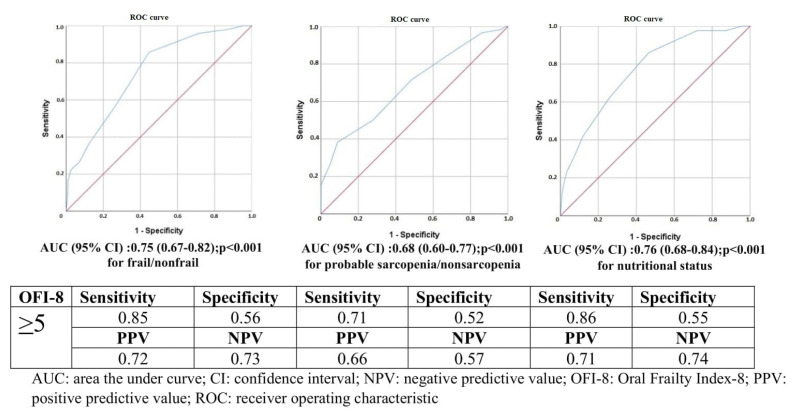
ROC analysis of the OFI-8.

In Turkish older adults, the ROC curves for cut-off values of physical frailty, nutritional status, and probable sarcopenia based on the OFI-8 total score are shown in Figure 2. Accordingly, for these three conditions, the OFI-8 total score cut-off value was determined as ≥5 (sensitivity for physical frailty is 85%; sensitivity for malnutrition is 86%; sensitivity for probable sarcopenia is 71%). The area under the curve (AUC) values were 0.75 (95% CI 0.67–0.82) for physical frailty, 0.76 (95% CI 0.68–0.84) for malnutrition, and 0.68 (95% CI 0.60–0.77) for probable sarcopenia.

### 3.6. Oral frailty status

When participants with an OFI-8 total score of 5 or higher were considered oral frail, the prevalence of oral frailty was 57.1%. When the patients were divided into two groups as oral frail and oral nonfrail, the rate of female sex, age, frequency of chronic lung disease, geriatric syndromes including recurrent falls, probable sarcopenia, physical frailty, and malnutrition were observed to be statistically significantly higher in the oral frail group compared with the oral nonfrail group (p < 0.05) (Table 5). When adjusted for demographic characteristics, including age and sex, in a binary logistic regression analysis, the significance of probable sarcopenia, physical frailty, polypharmacy, malnutrition, and falls remained (p < 0.05) (Table 6). There is no multicollinearity.

## 4. Discussion

This study demonstrated that the OFI-8 is a reliable and valid instrument for evaluating oral frailty among Turkish older adults. It also demonstrated that the OFI-8 has high sensitivity and specificity, test–retest reliability, concurrent validity, and internal consistency in distinguishing patients with oral frailty. Furthermore, the Turkish version of the tool is correlated with muscle strength, physical frailty, malnutrition, and polypharmacy in the same population, with the cut-off point of 5.

Recent evidence suggests that frailty may be evaluated across multiple subdomains. One of these domains, oral frailty, is a newly defined geriatric syndrome characterized by age-related decline in oral function, including tooth loss, poor oral hygiene, inadequate dental prostheses, difficulty chewing and swallowing, and a concomitant decrease in cognitive and functional abilities [13,23]. It has been reported that deterioration in oral health is associated with age-related physiological burden, physical frailty, sarcopenia, cognitive impairment, and multimorbidity in older individuals [23]. Therefore, considering its reversibility and negative health consequences, oral frailty (OF) is of great importance to screen for older individuals as a component of the comprehensive geriatric assessment [13,24]. In this particular concept, various tools have been developed to evaluate OF. Among them, the most popular is the OFI-8, developed by Tanaka et al. following the establishment of the Oral Frailty Index-6 [15]. The latter is difficult for health professionals to apply, whereas the former is easy to use. In this study, the Turkish version of the tool demonstrated reliability and validity for evaluating oral frailty and adequate sensitivity and specificity for distinguishing patients with oral frailty.

Several validation studies of the OFI-8 have been conducted across different linguistic and cultural contexts, including Japanese, Chinese, English, and Portuguese. The original Japanese version demonstrated good internal consistency and predictive validity for physical frailty and disability in community-dwelling older adults [15]. The Chinese validation confirmed strong associations between OFI-8 scores, chewing ability, dentition status, and nutritional risk, showing good reliability and construct validity [25]. The English adaptation has been used to explore oral frailty in Western populations, supporting its clinical and cross-linguistic applicability [10]. Recently, a Portuguese validation study further confirmed the OFI-8’s reproducibility and internal consistency, emphasizing its feasibility in Latin cultural settings [26]. However, before the present research, no validated Turkish or Middle Eastern adaptation existed, despite substantial linguistic and cultural differences in dietary patterns, oral health behaviors, and healthcare accessibility. By providing the first Turkish version, this study fills an important cross-cultural gap and extends the global utility of the OFI-8 in geriatric screening and research.

In addition, the prevalence of oral frailty was reported to range from 33.9% to 59.2% [25]. This difference in frequency may vary depending on the nature of the sample in the studies, the location where the study was conducted (community, hospital, nursing home), and socioeconomic characteristics. In accordance with the extant literature, the frequency was 57.1% in ours. To clarify this variation, population-based studies are also needed to estimate the true prevalence.

Moreover, because oral health-related behaviors vary significantly across cultures, the content of the tool can differ between societies. The recent study emphasizes that poor oral health is highly prevalent among older adults in Türkiye. It identifies key contributing factors such as low socioeconomic status, limited education, and inadequate access to dental care services [27]. Recent Turkish epidemiologic data have demonstrated that oral health disparities are strongly associated with lower education levels, socioeconomic inequalities, and limited accessibility to preventive dental services [27–29]. Furthermore, recent national policy analyses emphasize the need for an organized oral health framework to enhance accessibility and preventive service delivery across regions, particularly in rural areas. These findings highlight the need to address social and healthcare inequalities to improve oral health outcomes among older adults in Türkiye. Concordantly, use of dentures, irregular dental visits, and inadequate tooth-brushing habits are key items of the OFI-8. It is also quite remarkable that 91.9% of the population were using dental prostheses in the present study. Therefore, it is essential to emphasize that primary care physicians and dentists play a crucial role in maintaining oral health among elderly individuals, thereby protecting their overall well-being. Additionally, inadequate dental care may be associated with numerous adverse health consequences, ranging from periodontal disease to pneumonia [30].

Each community must determine a specific cut-off value for a diagnostic or screening tool to distinguish between normal and abnormal. In light of this information, the cut-off value for OFI-8 was determined as ≥5, with high sensitivity for physical frailty, malnutrition, and probable sarcopenia in Turkish older adults, which differs from the ≥4 reported by Tanaka [15]. In the present study, the optimal cut-off value for oral frailty was determined as ≥5, which is slightly higher than the ≥4 reported by Tanaka et al. in Japanese community-dwelling older adults. This variation may reflect differences in cultural and dietary habits, oral health behaviors, and access to dental care services across populations. In fact, given the variables playing a role in the development of Oral frailty, such as limited food variety, secondary low fiber intake, decreased chewing ability, dysphagia [31], tongue pressure, oral dysdiadochokinesia [32], and dental care, some of which may be specific to each population, the disparity between ours and Tanaka’s is not surprising. Moreover, it has been shown that OF is associated with falls, low dietary diversity, social withdrawal, long-term care needs, cognitive dysfunction, and premature mortality, in addition to physical frailty, sarcopenia, and malnutrition [8,33,34], as observed in our study.

Oral frailty is also associated with an age-related decrease in chewing ability and oral motor skills. Chewing ability in the elderly decreases due to tooth loss and impaired tongue motor functions [35]. This study emphasized that oral frailty is associated with recurrent falls across age and sex, as reported in the extant literature. Furthermore, diminished masticatory ability and tooth loss play a central role in the development of oral frailty [31,35]. Tooth loss reduces occlusal support and masticatory efficiency, leading to the avoidance of hard foods and a preference for soft, low-fiber diets, which may accelerate malnutrition and sarcopenia [31,32]. Reduced chewing ability is also associated with decreased tongue pressure and oral motor coordination, contributing to swallowing difficulty and decreased dietary diversity [32]. In addition, masticatory function has been shown to correlate with physical performance and balance control, potentially mediating the link between oral frailty, frailty, and recurrent falls [34]. The preservation of chewing ability through proper dental care and prosthetic rehabilitation is therefore essential for maintaining nutritional status and preventing further functional decline in older adults [35]. Age-related dry mouth or xerostomia is another factor that may affect oral health in the elderly [31]. Age-related dry mouth, resulting from decreased saliva secretion, leads to adverse outcomes such as taste disturbances, halitosis, oral pain, dysphagia, articulation disorders, increased oral infections, and caries/demineralization [31], as well as drug-related concerns, such as polypharmacy and anticholinergic burden [36, 37]. This study, as in previous ones, found that the number of medications was correlated with the total oral frailty score and that polypharmacy frequency was higher in the oral frailty group. In clinical practice, the OFI-8 can be easily administered by primary care physicians, nurses, or dentists as part of routine geriatric screening. Its short, self-reported structure allows rapid assessment of oral frailty, facilitating early detection of individuals at risk of malnutrition, sarcopenia, and physical frailty in community and outpatient settings in Türkiye.

The study has several strengths. To the best of our knowledge, it is the first Turkish validation study of the OFI-8. Additionally, oral frailty has been shown to be associated with physical frailty, probable sarcopenia, recurrent falls, polypharmacy, and malnutrition. This study has several limitations to be considered. First, its cross-sectional design precludes establishing a causal relationship between oral frailty and associated geriatric syndromes. Second, as the study was conducted in a single tertiary geriatric outpatient clinic, the findings may not be fully generalizable to community-dwelling older adults or those from different geographic or socioeconomic backgrounds. Third, although EFA was conducted to examine construct validity, confirmatory factor analysis was not performed to validate the two-factor structure. Finally, individuals with moderate to advanced dementia were excluded because of their inability to self-report, which may limit the generalizability of the findings to this vulnerable subgroup of older adults.

In conclusion, the Turkish version of the OFI-8 is a valid, reliable, and easy-to-use tool for defining oral frailty in Turkish older adults. Given the potential complications and comorbidities associated with oral frailty, it is imperative to acknowledge that timely recognition and treatment of oral frailty in elderly individuals may facilitate its management and associated comorbidities, particularly frailty, sarcopenia, and malnutrition, and enhance patients’ quality of life. Routine oral frailty screening using the OFI-8 may facilitate early identification of individuals at risk for sarcopenia and malnutrition, thereby guiding multidisciplinary interventions in geriatric clinics.

## Figures and Tables

**Table 1:** Original and Turkish versions of the OFI-8.

Item	Original version (English)	Translation (Turkish)
1	Do you have any difficulties eating tough foods compared to 6 months ago?	6 ay öncesine kıyasla katı gıdaları yemekte zorlanıyor musunuz?
2	Have you choked on your tea or soup recently?	Son zamanlarda çay ya da çorba içerken soluk borunuza kaçtı mı?
3	Do you use dentures?	Diş protezi kullanımı var mı?
4	Do you often have a dry mouth?	Sık sık ağzınız kurur mu?
5	Do you go out less frequently than you did last year?	Geçen yıla göre daha mı az evden dışarı çıkarsınız?
6	Can you eat hard foods like squid jerky or pickled radish?*	Havuç, turp, kuru ekmek gibi sert yiyecekleri yiyebilir misiniz?*
7	How many times do you brush your teeth in a day? (2 or more times/day)	Dişlerinizi günde kaç kez fırçalıyorsunuz? (günde 2 veya daha fazla)
8	Do you visit a dental clinic at least annually?	En az yılda bir kez diş hekimine başvurdunuz mu?

* You can diversify these foods according to regional differences.

* Bu gıdaları yöresel farklılıklara göre çeşitlendirebilirsiniz.

**Table 2a:** Item–total correlations and Cronbach’s alpha if item deleted (OFI-8).

Item no	Item label	Corrected item–total correlation	Cronbach’s α if item deleted
**1**	Eating tough foods	0.496	0.704
**2**	Choking	0.448	0.725
**3**	Denture use	0.316	0.741
**4**	Dry mouth	0.349	0.746
**5**	Decreased going out	0.561	0.680
**6**	Eating hard foods	0.501	0.701
**7**	Tooth brushing ≥2/day	0.366	0.737
**8**	Dental visit annually	0.310	0.739

**Table 2b:** The test–retest reliability of the OFI-8.

		95% CI		*F* test	
	Intraclass correlation	Lower bound	Upper bound	Value	*p-value
Single measures	0.924	0.87	0.95	25.56	**<0.001**
Average measures (two measures within 14 days)	0.961	0.93	0.97	25.56	**<0.001**

* Statistically significant p-values (p < 0.05) are shown in bold characters.

**Table 3:** EFA: factor loadings and explained variance of the OFI-8.

Item no.	Item label	Oral function component	Oral health behavior component
**1**	Eating tough foods	0.77	—
**2**	Choking	0.71	—
**3**	Denture use	—	−0.59
**4**	Dry mouth	0.59	—
**5**	Decreased going out	0.52	−0.53
**6**	Eating hard foods	0.7	—
**7**	Tooth brushing ≥2/day	—	−0.68
**8**	Dental visit annually	—	−0.74

Explained variance: oral function component = 27.8%, oral health behavior component = 21.9%; total = 49.7%

**Table 4:** Correlation of OFI-8 total test scores with other parameters.

	r correlation coefficient	Correlation strength	p-value
Number of medications	0.335	low	**<0.001**
CCI	0.196	low	**0.013**
MNA-SF score	−0.435	moderate	**<0.001**
Fried Frailty Index	0.534	moderate	**<0.001**
Muscle strength (kg)	−0.450	moderate	**<0.001**
Basic ADLs	−0.551	moderate	**<0.001**
Instrumental ADLs	−0.326	low	**<0.001**
TUG test duration (s)	0.523	moderate	**<0.001**
MMSE	−0.116	low	0.190
Yesevage 15-item GDS	0.243	low	**<0.001**

* Statistically significant p-values (p < 0.05) are shown in bold characters

ADLs: activities of daily living; CCI: Charlson Comorbidity Index; GDS: geriatric depression scale; MMSE: Mini-Mental State Examination; MNA-SF: Short-Form Mini-Nutritional Assessment

**Table 5:** Comparison of demographic features, comorbidities, and geriatric syndromes according to oral frailty status.

	Oral frailty (n = 92)	Oral nonfrailty (n = 69)	p-value*

***Demographic features***			

Age			
Mean ± SD	78.87 ± 7.47	75.45 ± 7.10	**0.006**
Median [IQR]	80 [11]	74 [12]	

Sex (%; female)	70.7	53.6	**0.026**

Education year			
Mean ± SD	5.50 ± 3.31	6.84 ± 3.52	0.106
Median [IQR]	5 [3]	5 [3]	

Living alone (%)	21.7	17.4	0.827

Married (%)	62.0	62.3	0.426

***Comorbidities (%)***			

Hypertension	74.7	66.7	0.265

Diabetes mellitus	39.1	37.7	0.852

Chronic cardiac diseases	46.2	34.8	0.148

Peripheral artery disease	4.3	7.2	0.428

Osteoporosis	21.7	13.0	0.155

Chronic lung diseases	25.0	11.6	**0.033**

CCI			
Mean ± SD	1.37 ± 1.16	1.04 ± 1.02	0.066
Median [IQR]	1 [2]	1 [2]	

***GERIATRIC SYNDROMES (%)***			

Recurrent falls (≥two in a year)	43.5	23.2	**0.007**

Urinary incontinence	46.7	46.4	0.964

Sleep disorder	46.7	37.7	0.250

Polypharmacy (≥five medicines)	73.9	53.6	**0.007**

Probable sarcopenia	46.7	24.6	**0.004**

Physical frailty	45.7	10.1	**<0.001**

Malnutrition	40.2	8.7	**<0.001**

Geriatric depression	39.1	36.2	0.708

CCI: Charlson Comorbidity Index; IQR: interquartile range; SD: standard deviation

* Statistically significant p values (p < 0.05) are shown in bold characters.

**Table 6:** Examining polypharmacy, recurrent falls, frailty, and probable sarcopenia according to oral frailty status using logistic regression analysis.

Variables	Odds ratio	95% CI	*p-value	Hosmer–Lemeshow test		Nagelkerke R^2^

				χ ^2^	p-value	

Recurrent falls						
Unadjusted	2.54	1.27–5.10	**0.007**			
Adjusted	2.73	1.30–5.72	**0.012**	4.96	0.761	0.176

Polypharmacy						
Unadjusted	2.62	1.32–5.22	**0.007**			
Adjusted	2.42	1.17–4.99	**0.033**	10.59	0.226	0.150

Probable sarcopenia						
Unadjusted	2.68	1.35–5.31	**0.004**			
Adjusted	3.99	1.79–8.86	**0.002**	9.11	0.332	0.215

Physical frailty						
Unadjusted	7.44	3.07–17.98	**<0.001**			
Adjusted	5.57	2.25–13.77	**<0.001**	3.94	0.862	0.242

Malnutrition						
Unadjusted	7.06	2.77–17.99	**<0.001**			
Adjusted	5.51	2.10–14.22	**0.001**	0.174	0.229	0.229

χ^2^: chi-square

* p-value was created by applying binary logistic regression adjusted for demographic characteristics, including age and sex.
